# A “Near-Miss Lethal Accident Case” in MR Suit of a Tertiary Care Hospital

**DOI:** 10.1155/2011/793570

**Published:** 2011-10-30

**Authors:** Samina Mufti, Mushtaq A. Sheikh, Abdul Hakim, Showkat A. Mufti, Farooq Jan

**Affiliations:** ^1^Department of Hospital Administration, SKIMS, Kashmir Srinagar 190011, India; ^2^Department of Internal and Pulmonary Medicine, SKIMS, Kashmir Srinagar 190011, India

## Abstract

A “near miss” is an unpleasant event that did not result in injury, illness, or damage but had the potential to do so, but for a fortunate break in the chain of events. We present a near-miss case which occurred in the MR suite of a tertiary care hospital. Although the MR is considered a very safe procedure, if MR safety guidelines are not adhered to, adverse and catastrophic events to the extent of patient deaths are known to have occurred. It is hoped that this incident will prompt hospitals to document and follow MR safety protocols for patient and staff safety. Although MRI is an extremely safe procedure rarely MR adverse incidents have resulted in serious physical injury or even death. The incident is an eye opener regarding potential adverse events lurking in the relatively safe MR environment and provides an opportunity to rectify the inadequacies in MR safety.

## 1. Introduction

A “near miss” is an unpleasant event that did not result in injury, illness, or damage but had the potential to do so, but for a fortunate break in the chain of events. A human error compounded with a faulty system invariably permits or compounds the harm and should be the focus of improvement. We present one such event in the MRI section of a tertiary care hospital with the aim of identification and correction of the loopholes in the system, to ensure safety of patients, their attendants, staff, and equipment installations. The case has its importance as very few such cases have been reported and literature review did not reveal reporting of any near-miss case.

## 2. Case History

**Figure 1:**
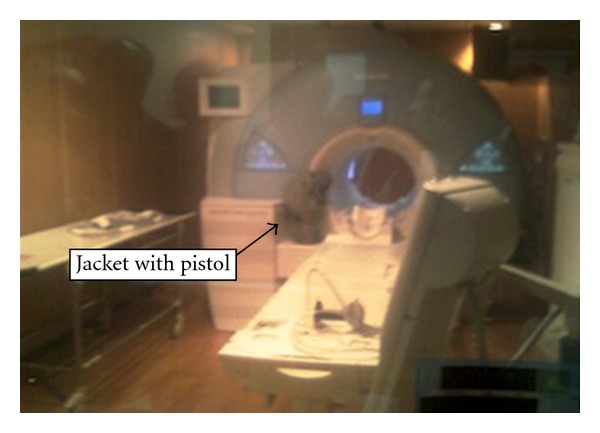
Photograph showing jacket with pistol caught in the magnetic bore.

A fifty-five-year-old man was taken up for MRI of the lumbosacral spine for detecting metastases from bronchogenic carcinoma of the lung. He was accompanied by his son (a government security officer) who was allowed with him for assistance in the MRI scanner room. Both the patient and accompanying person were verbally asked to remove all ferromagnetic unsafe objects, from their possession. The accompanying person wore a jacket with a pistol in the inside pocket, which was inadvertently forgotten and not removed. As a result when the patient's son neared the magnet, he felt a strong pull towards the magnet of the scanner. He was frightened and quickly pulled of his jacket which flew to the magnetic bore where it was lodged. The examination was abandoned at the time. Fortunately, the jacket could be removed along with the pistol by a careful and sustained pull, without quenching the magnet without damage to all the persons in the magnet room, computer room, or the equipment (see Figure 1). Otherwise, quenching the magnet would have been required as suggested by engineering department.

## 3. Discussion

**Figure 2:**
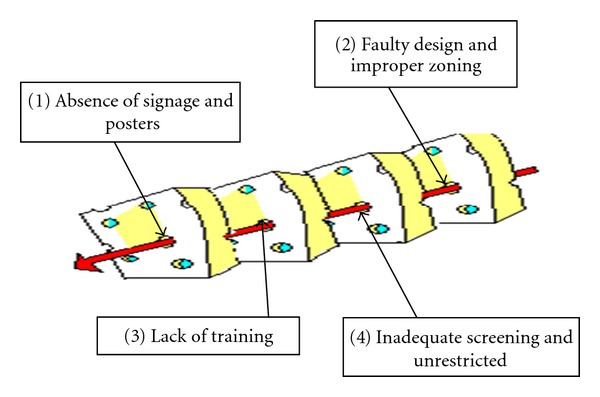
Swiss cheese model of the near-miss incident.

Accidents in the MR facilities have been reported in the literature and have occasionally proven lethal. According to a case report, a thumb locked firearm once discharged spontaneously in an MR section of a hospital [2]. Fortunately, none was injured and only minimal cosmetic damage occurred to the magnet. In the case under discussion, although the pistol of the officer was dual locked, the magnetic pull could have unlocked the pistol and a potentially fatal accident could have occurred. Chaljub et al. have presented cases of oxygen/nitrous cylinder turned projectile, accidents in the magnetic field vicinity of MR facilities [3]. These incidents resulted in huge financial liabilities, either as legal compensation to the patient (one patient sustained a facial fracture) or as repair and restoration of the MR equipment. These events which led to the near-miss accident in the tertiary care hospital are discussed hereunder and depicted in the Swiss Cheese Model (Figure 2).

These were (a) faulty design and consequent improper zoning of the MRI section of the hospital, (b) unrestricted access of patients and their attendants, (c) incomplete screening partly due to (d) inadequate training of the personnel, including doctors and technologists, and (e) absence of signage and posters. When all the holes signifying the sequence of events lined up in the cheese, the “near-miss” incident occurred.

**Figure 3:**
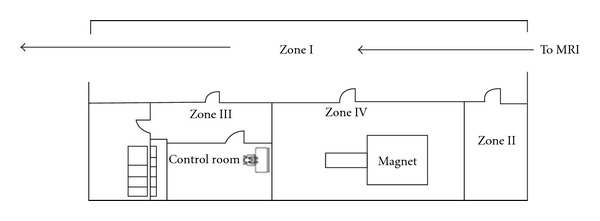
Floor plan of the MRI facility of the hospital.

The design of the MRI facility of the hospital in which the near-miss event occurred does not conform to the standards. As a result, the zones of the facility lack a functional flow pattern. Entry to zone 4 (MR Scanner Room) directly opens to the corridor (zone I) of the Department of Radio-diagnosis and Imaging, through which patients and their attendants approach the CAT scan facility. Whenever the MR scanner door opens, a potential catastrophe awaits as critical patients on portable oxygen cylinder traverse through this corridor while being taken for CT scan (Figure 3).

**Figure 4:**
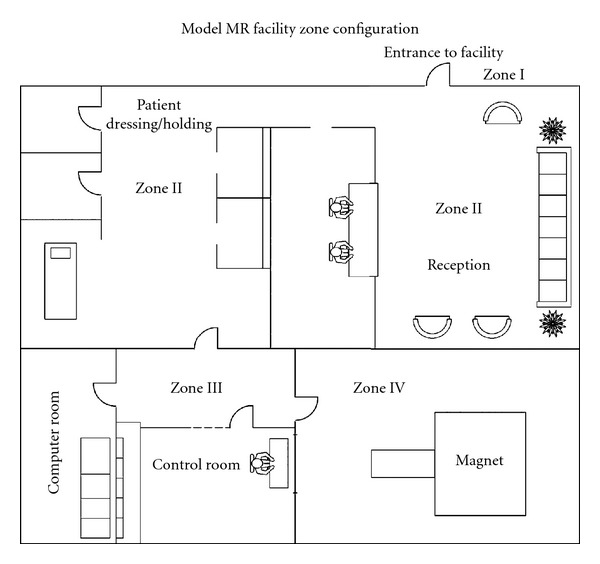
Idealized sample floor plan illustrates site access restriction considerations [1].

 Incidents have been reported when oxygen cylinders turned into projectiles, once they were inadvertently brought into the vicinity of the magnetic field. One fatality, many injuries and damage to the magnet have thus been reported [3, 4]. According to the ACR Guidance Document for Safe MR practices: 2007, the facility should be designed so that proper zoning is maintained. The zones should have a functional relationship so that entry happens only from the lower to a higher zone (Figure 4).

 Non-MR Personnel including attendants of patients enter zone III of the hospital's MR facility, when there are strict guidelines about site access restriction to MRI facility. According to the ACR Guidance Document-2007, the patient and the accompanying person (if absolutely needed) are under strict supervision of level I and level II MR persons in zone III, and level II MR person in zone IV. The supervision is either direct visual or through audiovisual monitoring [1].

 The screening form is inadequately filled by the referring physician. The patient is not required to fill the form, nor is the form translated into locally used languages. Although the patient is interviewed by the MR physician regarding any metal implant or related contraindications and precautions, a metal detector is seldom used although a ferromagnetic detector is available. Similarly, if an attendant must accompany a patient, an adequate screening is not performed.

Guidelines recommend that screening of the patient be performed in zone II by two individuals; one of these should be a level II MR person. The screening should be verbal interactive as well as written [5–7]. Instead of conventional airport type detectors, only ferromagnetic detectors should be used in the MR facilities [8, 9].

Only the faculty of the Department of Radiodiagnosis have been trained at the time of installation of the equipment, that is, five years back. Therefore, in reality, all the personnel working in the MRI section are non-MR personnel. ACR guidelines stress that personnel including radiologists and technologists must undergo training every year to qualify as MR personnel [1].

 Although hazard signs are posted at the entry of zone IV of the MR section, the red light signifying that the “magnet is on” is not visible at the site, as a result of which no caution is affected. Posters for education and awareness of public are conspicuously absent. A gauss line has not been drawn; therefore, the magnetic effect in the vicinity is unknown.

Furthermore, an MR safety manual is not available and hence not used. Policies and procedures regarding safety in the MR environment are not clearly laid down.

## 4. Conclusion

Although MRI is an extremely safe procedure if MR safety instructions are adhered to and MR safe policies and procedures are followed, rarely MR adverse incidents have resulted in serious physical injury or even death. The incident was an eye opener regarding potential adverse events lurking in the relatively safe MR environment and provides an opportunity to rectify the inadequacies in MR safety. It is recommended that guidelines should be followed, MR safety manual developed and used. Adequate screening and warning systems should be established. A system of reporting of the near-miss/adverse incidents should be established in the hospital [10].
